# Subdivisions of the Auditory Midbrain (N. Mesencephalicus Lateralis, pars dorsalis) in Zebra Finches Using Calcium-Binding Protein Immunocytochemistry

**DOI:** 10.1371/journal.pone.0020686

**Published:** 2011-06-20

**Authors:** Priscilla Logerot, Nils O. E. Krützfeldt, J. Martin Wild, M. Fabiana Kubke

**Affiliations:** Department of Anatomy with Radiology, Faculty of Medical and Health Sciences, and Centre for Brain Research, University of Auckland, Auckland, New Zealand; University of Washington, United States of America

## Abstract

The midbrain nucleus mesencephalicus lateralis pars dorsalis (MLd) is thought to be the avian homologue of the central nucleus of the mammalian inferior colliculus. As such, it is a major relay in the ascending auditory pathway of all birds and in songbirds mediates the auditory feedback necessary for the learning and maintenance of song. To clarify the organization of MLd, we applied three calcium binding protein antibodies to tissue sections from the brains of adult male and female zebra finches. The staining patterns resulting from the application of parvalbumin, calbindin and calretinin antibodies differed from each other and in different parts of the nucleus. Parvalbumin-like immunoreactivity was distributed throughout the whole nucleus, as defined by the totality of the terminations of brainstem auditory afferents; in other words parvalbumin-like immunoreactivity defines the boundaries of MLd. Staining patterns of parvalbumin, calbindin and calretinin defined two regions of MLd: inner (MLd.I) and outer (MLd.O). MLd.O largely surrounds MLd.I and is distinct from the surrounding intercollicular nucleus. Unlike the case in some non-songbirds, however, the two MLd regions do not correspond to the terminal zones of the projections of the brainstem auditory nuclei angularis and laminaris, which have been found to overlap substantially throughout the nucleus in zebra finches.

## Introduction

The nucleus mesencephalicus lateralis pars dorsalis (MLd) has been extensively studied both anatomically and electrophysiologically in several avian species, e.g., chicken [1]–[5], guinea fowl [6], mallard [7], barn owl [8]–[15] and pigeon [16]–[18]. Fewer data are available for the MLd of songbirds, which comprise roughly half the number of avian species. In birds generally the nucleus is a major relay in the ascending auditory pathway to the forebrain but in songbirds it also has the special function of mediating auditory feedback for the learning and maintained production of song. We have previously reported on the ascending projections to MLd [19], [20]; in the present paper we describe the regional organization of MLd in zebra finches based on the different staining patterns resulting from the application of antibodies to three calcium binding proteins.

Most of our knowledge of the anatomical organization of MLd in songbirds derives from histochemical and immunohistochemical studies in zebra finches [21]–[24], and from studies that have examined auditory and somatosensory connections of MLd and adjacent structures in various finches [19], [20], [25], [26]. Here we define MLd as the midbrain region receiving ascending projections from the auditory brainstem, as previously described [19], [20]. In the midbrain of the zebra finch, as in other avian species, MLd is located within the intercollicular complex (ICo). Its boundaries can be defined on the basis of its cytoarchitecture, although its ventral and ventrolateral borders can prove difficult to differentiate from the adjacent ICo. MLd was originally shown by Karten [27] (see also [28]) in pigeons to project to the thalamic nucleus ovoidalis (Ov), a finding substantiated in ring doves [29], barn owls [30] and zebra finches [19].

We chose to describe the internal structure of MLd using antibodies against calbindin (CB), calretinin (CR) and parvalbumin (PV), three calcium-binding proteins (CaBPs) that are expressed in different neuronal subpopulations in both the peripheral and central nervous systems, with only a partial overlap [31], [32]. They belong to the EF-hand family of CaBPs [33] and generally bind to calcium ions (Ca^2+^) [34]. The expression of these three proteins has been used for many years to describe anatomically various sensory pathways and nuclei (for review see [31]), including the auditory torus of amphibians [35], [36], reptiles [37]–[40], birds [3], [5], [11], [12], [14], [21], [22] and mammals [41]–[57]. CaBP immunocytochemistry has been used previously to describe MLd in zebra finches [21], [22], although these studies mainly examined the patterns of expression in male birds (both adults and juveniles) and were performed in the absence of knowledge of the pattern of ascending inputs to MLd in this species. Here we extend these studies to describe the patterns of expression in adult male and female zebra finches, in the context of our knowledge of the ascending inputs to MLd [19], [20] and the electrophysiological in vivo data now available [58]–[62].

## Materials and Methods

### Animals

The experimental procedures were carried out according to the guidelines of, and were approved by, the Animal Ethics Committee of the University of Auckland (approval #R689). Birds were obtained from a local breeder, housed in a large flight aviary, provided with food and water *ad lib*, and maintained under a constant light/night cycle in the University of Auckland animal facility.

### Immunocytochemistry

Each of the 7 birds was deeply anaesthetized with an intramuscular injection of ketamine (Parnell Laboratories, Auckland, New Zealand; 100 mg/kg) and xylazine (Rompun Bayer; 20 mg/kg) and transcardially perfused with 0.9% saline followed by 4% paraformaldehyde in phosphate buffer (PB 0.1 M, pH 7.4) as per University of Auckland Animal Ethics Committee permits. The brains were post-fixed in 4% paraformaldehyde (PFA) before being cryoprotected in 30% sucrose in phosphate buffered saline (PBS 0.01 M, pH 7.4). They were then cut coronally on a freezing microtome and serial, free-floating 35 µm thick sections were collected in PBS in four columns. Sections in one column were mounted on subbed slides and stained with cresyl violet for the identification of cytoarchitecture. Sections in the three other columns were immediately processed for immunocytochemistry. After three 10 minutes rinses (all rinses in this protocol lasted 10 min) in PBS, endogenous peroxidase activity was blocked using 50% methanol and 1% hydrogen peroxide in distilled water for 10 min at room temperature. Sections were then rinsed 3 times in PBS before being incubated in a primary antibody against PV, CB or CR (monoclonal mouse anti-parvalbumin antibody: SWANT clone 235, Basel, Switzerland; monoclonal mouse anti-calbindin D28k: SWANT clone 300, Basel, Switzerland; polyclonal rabbit anti-calretinin antibody: SWANT 7699/4, Basel, Switzerland for 3 males and 3 females; monoclonal mouse anti-calretinin antibody: SWANT 6B3, Basel, Switzerland for one male) at a final dilution of 1∶5000 in PBS with 0.4% Triton X-100 (PBS-T) and 2% Normal Horse Serum (NHS) overnight at room temperature. After 3 rinses in PBS, sections were transferred to a biotinylated donkey anti-mouse or anti-rabbit secondary antibody (711-065-150 and 711-065-152, Jackson Immunoresearch Laboratories, Inc., West Grove, PA) in PBS-T with 2% NHS at a dilution of 1∶300 for 90 minutes at room temperature and rinsed 6 times in PBS before being incubated in avidin-biotin peroxidase complex (PIE 31001, Global Science and Technology Ltd, Auckland, New Zealand) at 1∶1000 in PBS-T for 1 h at room temperature. Following 6 rinses in PBS, sections were finally incubated in a chromagen-solution consisting of 0.025% 3,3′-diamino-benzidine (DAB), 0.005% H_2_O_2_ and 0.015% CoCl2 in PBS. The reaction was stopped by several washes in PBS. Sections were subsequently mounted on subbed slides, dehydrated in successive ethanol baths, cleared in xylene, and coverslipped using DePeX (Serva, Heidelberg, Germany).

### Analysis

All of the material was examined using light microscopy. Comparisons between different staining patterns were performed on adjacent sections, and the overall staining patterns and their boundaries were mapped onto a single, serially cut, cresyl violet-stained zebra finch brain for inter-individual comparisons. Outlines of the nucleus and visible subdivisions, were drawn using a camera lucida and then scanned into a personal computer for digital representation. Sections were also digitally photographed using a Nikon 80i light microscope (5 megapixel camera). Levels were then adjusted using Adobe Photoshop CS4 (Adobe Systems, San Jose, CA).

## Results

In the transverse plane MLd has an ovoid shape and is oriented obliquely along a ventrolateral to dorsomedial axis. At its caudal and rostral poles MLd is confined to a position laterally adjacent to the medial edge of ICo, but at intermediate rostrocaudal levels, where it reaches its maximum size, it expands laterally from this edge towards the tectal ventricle, from which it is separated only by a thin periventricular lamina [3]. At caudal levels MLd presents a dorsomedial extension called CM [caudomedial, 3], which has been identified in greenfinches [25], pigeons [18] and chickens [3]. At more rostral levels the dorsomedial border of MLd is straight-edged, where it abuts a core nucleus of the intercollicular complex [3], commonly known as the dorsomedial nucleus (DM). Unlike in pigeons [63] or chickens [64], MLd in zebra finches lacks a clear hilar region on its medial aspect [63], [64], so that its afferents from auditory brainstem nuclei enter the nucleus over a wide dorsoventral extent [19], [20].

### Parvalbumin-LI

**Figure 1:**
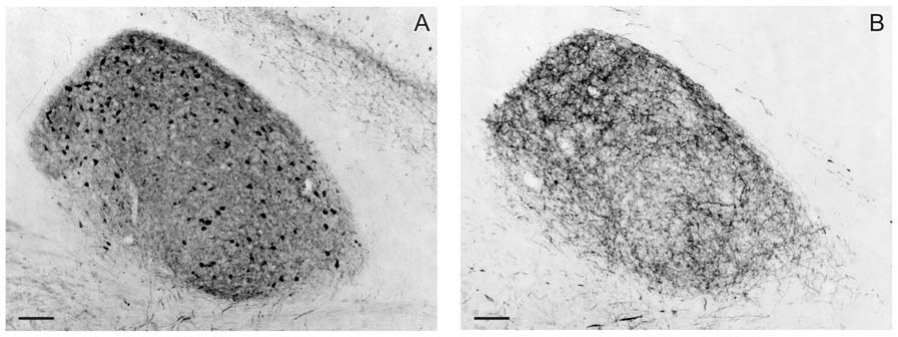
Pattern of parvalbumin-like immunoreactivity (PV-LI) and auditory terminal fields in MLd. Coronal sections through MLd in two different birds showing the correspondence between the PV-LI (A) and the ascending auditory terminal field obtained from injections to LLV (B). [Image in B was part of 20]. Scale bars = 100 µm.

**Figure 2:**
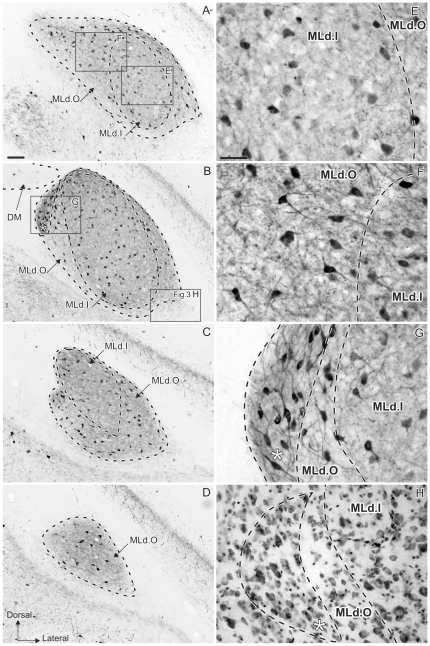
Patterns of parvalbumin-like immunoreactivity (PV-LI). **A–D:** caudal (top) to rostral (bottom) coronal sections showing PV-LI through the right MLd of a female zebra finch delineating two regions within the nucleus: MLd.O and MLd.I. The asterisk in **B** indicates the small nucleus within MLd.O that shows a different staining pattern. (DM is outlined for comparison with Figure 6.). **E–G:** high magnifications views of areas boxed in **A** and **B**. **E:** Photomicrograph of MLd.I (inset **E** in panel **A**) showing the specific neuropil staining and low punctuate density of this region. **F:** Photomicrograph of MLd.O (inset **F** in panel **A**) depicting the neuropil staining, high punctuate density and somata with visible processes specific to this region. **G:** high magnification view of the region marked by an asterisk in **B**. Note the darker neuropil staining than that of MLd.O and the absence of punctuate staining. **H:** Photomicrograph of a Nissl stained section also shown in Figure 5B, showing the dorsomedial MLd subnucleus marked with an asterisk in **B**. Scale bar: A–D = 100 µm; E–G = 50 µm.

The general parvalbumin staining pattern in MLd described by us [19] was shown to overlap closely the area in receipt of ascending brainstem auditory afferents, thereby defining the boundaries of MLd (Figure 1). Here we describe regional differences in the pattern of staining in more detail. Two regions within MLd were defined by the PV-LI: inner (MLd.I) and outer (MLd.O) (Figure 2, A–D). MLd.O was characterized by a PV-LI positive neuropil and by the presence of stained fibers and stained and unstained somata (Figure 2, F). (For descriptive purposes, we differentiate between staining of fibers within the neuropil and the rest of the neuropil.) The neuropil in this region showed a dense punctate staining pattern, whereas many of its neurons presented stained processes and those along the lateral edge of MLd exhibited elongated somata concentric with the overlying ventricle (not shown). At the level of DM, a small region on the dorsomedial aspect of MLd that can be identified in Nissl stained sections (Figure 2, B-asterisk, G, H-asterisk) exhibited a staining pattern distinct from that of the MLd.O. This small region had a darker PV-LI positive neuropil, stained fibers and stained somata and processes, but was characterized by an absence of punctate staining.

The inner region (MLd.I) could be distinguished by an apparent lower density of stained puncta (Figure 2, E; cf Figure 2, F). Like MLd.O, MLd.I also showed an immunopositive neuropil, stained and unstained somata, and some punctate staining. The processes of the stained neurons were, however, not visible in most cases. Incoming fibers - presumably mostly afferents from lower auditory nuclei - were seen mainly through the caudal half MLd. Caudally they were found widely dispersed along the ventromedial edge of the nucleus, while rostrally these were found more restricted to the most ventral edge (data not shown, but see [19], [20]).

### Calretinin-LI

**Figure 3:**
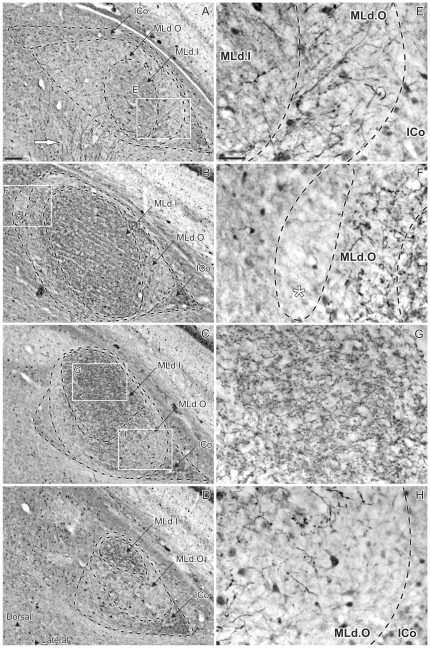
Patterns of calretinin-like immunoreactivity (CR-LI). **A–D:** caudal (top) to rostral (bottom) coronal sections showing CR-LI through the right MLd of a male zebra finch delineating MLd.I and MLd.O regions within the nucleus and the ICo as well as the trajectory of presumptive ascending auditory fibers (white arrow in A). The asterisk in **B** indicates the small nucleus within the MLd.O that shows a different staining pattern. **E–G:** high magnifications views of areas boxed in **A**, **B** and **C**. **E:** Photomicrograph of the MLd.I and MLd.O (inset **E** in panel **A**) showing the specific neuropil staining of these regions. **F:** high magnification view of the region marked by an asterisk in **B**. Note the lighter neuropil staining. **G:** Photomicrograph of MLd.I (inset **G** in panel **C**) showing the intense CR-LI staining of the neuropil. **H:** Photomicrograph of the MLd.O (inset **H** in panel **C**) showing the absence of neuropil staining and the presence of a few stained somata and fibers. Scale bar: A–D = 100 µm; E–G = 50 µm.

Brains of 3 males and 3 females were immunostained using a polyclonal anti-calretinin antibody and 1 other brain from a male was immunostained using a monoclonal anti-calretinin antibody. The staining patterns resulting from use of the monoclonal antibody were better delineated than those from use of the polyclonal, but were otherwise identical and are thus described together. Incubation with antibodies raised against calretinin resulted in a pattern of staining that, as in the case of parvalbumin, revealed two distinct regions within MLd: inner (MLd.O) and outer (MLd.I) (Figure 3, A–D). Caudally, MLd.O occupied a ventral position within the ICo, expanding towards the tectal ventricle at intermediate rostrocaudal levels (Figure 3, A). MLd.O lay on ventrolateral to dorsomedial axis, finally to be located in a dorsal position, underlain by the ICo at more rostral levels. The outer region presented a relatively unstained neuropil, within which some stained somata as well as numerous fibers were observed (Figure 3, E, H). Within this region, the same small, dorsomedial part found to be PV-positive (see above and Figure 2B, G) was either devoid of staining or presented a very light neuropil staining. (Figure 3, B-asterisk, F-asterisk).

MLd.I was readily distinguished from MLd.O by its darkly-stained neuropil (Figure 3, E, G). Some stained somata, as well as perikaryal ‘ghosts’ and terminal boutons, were observed. MLd.I occupied a central position caudally (Figure 3, A), extended along a ventrolateral to dorsomedial axis at intermediate levels where MLd is at its largest (Figure 3, B and C), and occupied a dorsal position rostrally (Figure 3, D). CR-LI fibers were seen running along the external surface of MLd.I, and some CR-LI fibers were also seen traversing it (Figure 3, E). The trajectory of presumptive ascending afferent fibers was seen mainly through the caudal half of the nucleus. As with the fibers showing PV-LI, CR-LI fibers entered MLd caudally from its internal edge over a wide extent (Figure 3, A), while rostrally they were restricted more ventrally.

The outer and inner regions defined by CR-LI correspond to the outer and inner regions defined by PV-LI. Outside but immediately adjacent to MLd, an external CR-LI positive region was identified (Figure 3, A–D), characterized by an immunopositive neuropil, within which numerous small stained somata were observed; a few of these also showed stained processes. Caudally and rostrally, this region surrounded MLd (Figure 3 A, C and D) and at intermediate levels it was restricted to a ventrolateral position (Figure 3 B). Since this region does not receive ascending auditory inputs [19], [20] it is considered part of ICo [19], [20].

### Calbindin-LI

**Figure 4:**
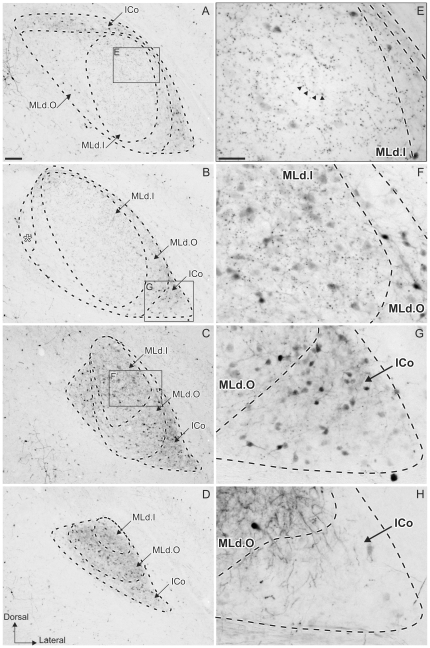
Patterns of calbindin-like immunoreactivity (CB-LI). **A–D:** caudal (top) to rostral (bottom) coronal sections showing CB-LI through the right MLd of a female zebra finch delineating MLd.I and MLd.O within the nucleus and the ICo. The asterisk in **B** indicates the small nucleus within MLd.O. Note that it is not as clearly visible as with the PV- and CR-like staining. **E–G:** high magnification views of areas boxed in **A**, **B** and **C**. **E:** Photomicrograph of MLd.I (inset **E** in panel **A**) showing the specific high density of labeled terminals in this region (some are indicated by arrow heads) and the absence of neuropil staining in intermediate sections. **F** Photomicrograph of MLd.I (inset **F** in panel **C**) showing the staining pattern in a more rostral section. Note the high density of labeled terminals and the difference in neuropil staining as well as the number of stained somata. **G:** Photomicrograph of ICo (inset **G** in panel **B**) showing the CB-LI staining of this region. **H:** Photomicrograph of PV-LI staining in ICo in an adjacent section (inset **H** in panel **B** – Figure 2) showing the absence of neuropil staining. Scale bar: A–D = 100 µm; E–G = 50 µm.

Calbindin-LI was also different in the inner (MLd.I) and outer (MLd.O) regions of MLd (Figure 4, A–D). At its most caudal and rostral levels, MLd showed a CB-LI positive neuropil and stained somata, some of which had immunostained processes and terminal boutons in both MLd.I and MLd.O (Figure 4, C and D). At intermediate levels, MLd.O showed light or no staining of neuropil, with some stained somata. The lateral and dorsal parts of MLd.O had a lightly stained neuropil, while its medial aspect appeared devoid of it (Figure 4, B). Also, the whole region presented some stained somata as well as a low density of terminal boutons. The subnucleus at the dorsomedial corner of MLd that shows distinct PV-LI and CR-LI and is also visible in Nissl stained-sections (Figure 4B asterisk), showed a light neuropil staining and some small stained somata with stained processes. In contrast to calretinin and parvalbumin staining, CB-LI did not stain presumptive afferent fibers to MLd. MLd.I did not present a homogeneous staining throughout the whole nucleus; at intermediate levels it was devoid of neuropil staining but was filled with a considerable number of terminal boutons and a few CB-LI positive large somata, which were less frequently observed than in MLd.O (Figure 4, A, B and E). Caudally and rostrally, however, the neuropil of MLd.I was stained and had greater density of somata (Figure 4, C, D and F). As in the CR-LI stained material, CB-LI was observed surrounding MLd caudally and rostrally, while occupying a ventrolateral position at intermediate levels (Figure 4, A–D). It was characterized by a CB-LI positive dark neuropil, some stained fibers and terminal boutons, and numerous small stained somata, some with stained proximal processes (Figure 4, G). This region is not the recipient of ascending auditory inputs and is therefore considered part of ICo rather than of MLd.

### Summary of staining patterns

**Figure 5:**
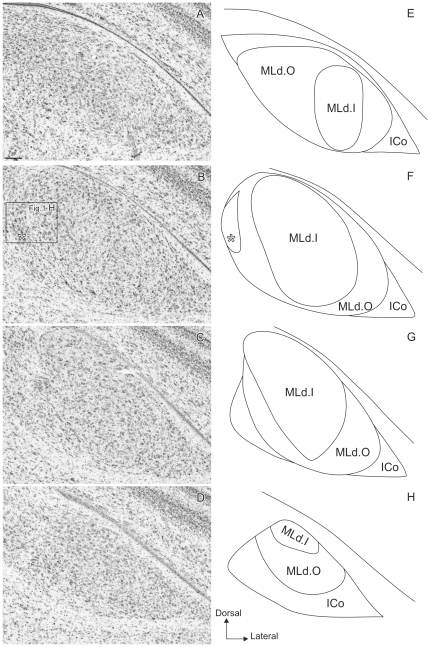
Subdivisions of MLd in Nissl stained material. **A–D:** caudal (top) to rostral (bottom) coronal Nissl-stained sections through the right MLd. The asterisk in **B** indicates the small nucleus within MLd.O showing a different staining pattern in PV- and CR-LI adjacent sections. Note that it is clearly visible in Nissl-stained sections. **E–G:** Schematic drawings of the different regions based on PV-, CR- and CB-LI staining. The asterisk in **F** indicates the subnucleus delineated by the PV- and CR- LI staining and by Nissl staining. Note how the boundaries can be identified by naked eye for more intermediate sections (**F** and **G** and corresponding panels **B** and **C** respectively) while they are obscure in the more caudal and rostral ones (**E** and **H** and corresponding panels **A** and **D** respectively). Scale bar = 100 µm.

PV staining was commensurate with the whole of MLd, defined as that region of the torus receiving direct and indirect ascending auditory afferents [19], [20]. Within the PV-LI region, two regions based mainly on the apparent abundance of puncta could be differentiated. An inner region, which had less punctate staining, corresponded to a region that also exhibited a CR-LI-positive neuropil and matched the part of MLd where most of the terminal boutons showing CB-LI were concentrated. We have termed this the inner region MLd.I. An outer region of MLd (MLd.O), which surrounded the inner region, was characterized by PV-LI exhibiting a higher density of punctate staining than that of MLd.I. In contrast, MLd.O exhibited a CR-LI negative neuropil, but was rich in CR-positive fibers and also presented a few CB-LI-positive somata and a light CB-LI positive neuropil. Surrounding MLd is a CB-LI- and CR-LI- positive region (Figures 3 and 4) which is not stained with PV (Figure 4, H), does not receive ascending auditory projections and is, therefore, considered part of ICo. It is interesting that the set of CaBPs used in the current study clearly delineate two regions within MLd, as well as the ICo (Figure 5, E–H), whereas these boundaries do not always appear as easily identifiable in Nissl stained material (Figure 5, A–D) especially at the most caudal and rostral levels (Figure 5, A and D).

**Figure 6:**
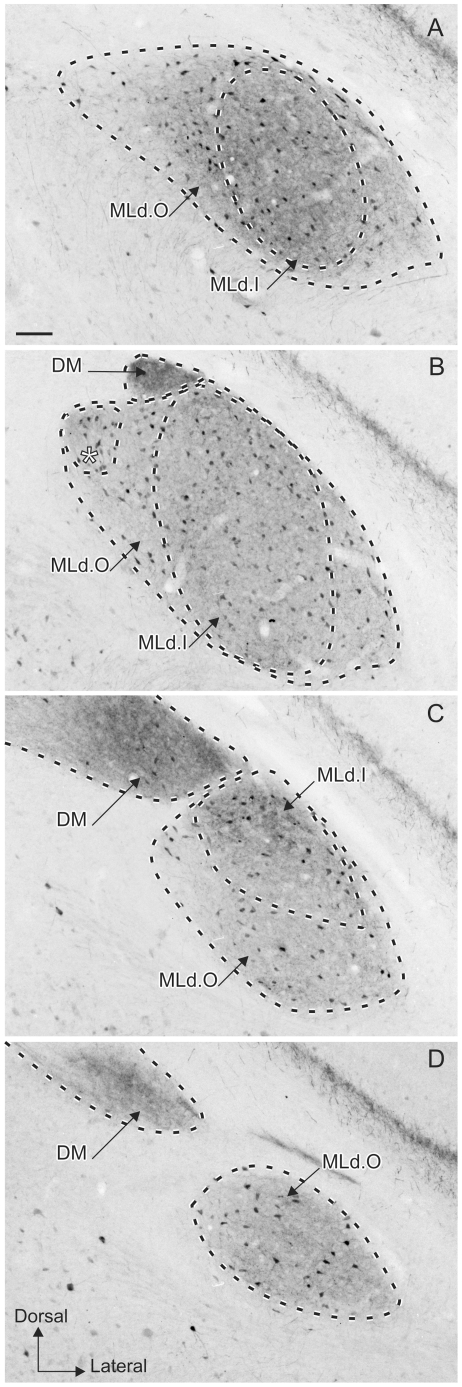
Parvalbumin-like immunoreactivity in males and females. **A–D:** MLdCaudal (top) to rostral (bottom) coronal sections showing PV-LI through the right MLd delineating MLd.I and MLd.O within the nucleus in a male zebra finch. A comparison with Figure 2 A–D shows the absence of sexual dimorphism in the staining of nucleus MLd and the presence of sexual dimorphism in the staining of nucleus DM (see text). Scale bar = 100 µm.

CaBP staining patterns revealed no differences between males and females in the organization of the auditory midbrain, except for differences in PV staining patterns of DM (compare Figure 2 A–D with Figure 6). We did not quantify cell numbers in MLd in our various cases, but no striking differences were apparent either between males and females (see Figures 2A–D and 6) or between left and right MLds.

## Discussion

We have used CaBP immunostaining to identify the regional organization of MLd within the context of its ascending projections recently described [19], [20]. An inner and an outer region of MLd (MLd.I and MLd.O, respectively) can be distinguished based on the patterns of CaBP immunoreactivity, which also distinguish MLd proper from its surrounding ICo. The patterns of staining in the areas examined in the midbrain were similar in males and females, with the exception of DM. In females, only few DM somata were immunostained (Figure 2, B), while in males DM showed a strong PV-LI neuropil with few stained somata (Figure 6, A–D), as shown previously in the context of song system anatomy [65]. This is interesting because zebra finches – and songbirds in general – are known to present sexual dimorphism of certain brain areas. Adult females, for instance, do not possess a song system (interconnected nuclei, distinct from the auditory pathway, involved in both vocal learning and vocal motor output) as developed as that seen in males [66]. DM receives direct descending projections from the forebrain robust nucleus of the arcopallium [67]–[70] in both sexes, but only in males do projection neurons in RA and their terminal fields in DM stain with PV [65]. Sexual dimorphism has also been shown in the higher auditory center NCM, where Pinaud et al. [71], found that males had twice as many CB-LI positive cells as females.

### Comparison with other avian species

Most of what is known about anatomical subdivisions of the avian MLd, based on cytoarchitecture and/or neurochemistry, ascending projections from the brainstem or neurophysiological properties, applies to non-songbirds (chicken: [1]–[5]; guinea fowl: [6]; mallards: [7]; owl: [8]–[15]; pigeon: [16]–[18]). In comparison, data available for songbirds are fewer [19]–[24], [58]–[62], [72], [73]. Recent studies [19], [20] have shown that the projection patterns of the ascending inputs to the auditory torus in zebra finches differ from those described for non-songbirds, thereby challenging the validity of a general schema for MLd subdivisions [3], [13], [14], as well as the generally presumed subdivisions of MLd in songbirds [21], [22], [73]. While the terminal fields of NA and NL projections are segregated within MLd in chickens [2], [4], pigeons [17], [18] and barn owls [13], they extensively overlap in zebra finches. Thus, segregated terminal fields within MLd cannot be used in the zebra finch (and presumably not in the closely related Bengalese finch) to differentiate what in other species are called core and shell. Braun et al. [21], [22] and Zeng et al. [73] have attempted to identify the regional organization of MLd in zebra and Bengalese finches, respectively. Braun and her colleagues described the complementary staining patterns of PV and CB (and other markers) in the brain of young male zebra finches and implied that the PV positive staining was restricted to a core of MLd, while a shelf (or shell) or marginal zone of MLd was characterized by CB-positive immunoreactivity. This differentiation, which has also been referred to (and presumably accepted) by other authors, we contend blurs the real boundary of MLd. Specifically, we suggest that the CB positive region that they seem to consider part of their shell is actually part of ICo, since it corresponds to a similar CB-positive region defined in the present study, but one that is now known not to receive ascending auditory afferents and therefore not part of MLd. If this is correct, the ‘core’ defined by PV-positive staining in the studies of Braun et al. would comprise the entirety of MLd.

Zeng et al. [73], using antibodies to Met-enkephalin, substance P and serotonin, suggested that the MLd of Bengalese finches was equivalent to MLd of barn owls. Thus, an MLd core and shell in Bengalese finches was considered analogous to the core and both lateral and medial shells of the central nucleus of the inferior colliculus of barn owls [13]. Zeng et al. also considered that ICo was equivalent to the external nucleus of the inferior colliculus of mammals. In their study, the core of MLd can easily be identified by a lack of substance P, as was reported for pigeons [18], but the boundary between shell and ICo remained quite unclear (see Figure 8, p. 11 in Zeng et al. [73]). The results of Zeng et al., when compared to ours, suggest that their subdivisions are congruent with ours, but a re-examination of their neurochemical boundaries with regards to known projection patterns needs to be reassessed. The complementarity of the CB and PV staining patterns characteristic of ICo and MLd, respectively, is, however, strikingly clear in their study on the neurogenesis of core and shell areas in the chick brain [5]. Still, neither of their two studies included the use of an antibody against calretinin, which Puelles et al. [3] suggest is the appropriate marker for the core region of the central nucleus in chickens, in which a dark CR-positive plexus appears to match the projections of NL onto MLd in this species [2], [4], as it does in barn owls [11], [12], [14]. Since we have now traced the ascending projections to MLd and described their terminal fields within the nucleus in zebra finches [19], [20], we can try to correlate these results with the subdivisions observed using CaBPs in the present study. In doing so, it appears that the PV-positive staining demarcates the terminal fields of ascending projections of both NA and NL and the nuclei of the lateral lemniscus, and should therefore include all MLd subdivisions. Based on this hodological criterion, the CB-positive external zone should not be considered part of MLd, but rather of ICo, which is in agreement with the organization proposed by Zeng and his colleagues for Bengalese finches [73]. Moreover, within the PV-positive region in the present study of zebra finch MLd, two distinct regions could be delineated based on the density of puncta. Also, the central zone with less puncta anatomically matched the dark CR-positive central neuropil within MLd. The two MLd regions, inner and outer, may, or may not, represent two functionally different zones. For instance, in barn owls, core and shell regions are the recipients of NL and NA projections, respectively, and have specific neurophysiological properties that originate from separate time and intensity pathways (referred as ITD and ILD, respectively). These pathways eventually converge in the lateral shell, from where projections are sent to the external nucleus, ICX, where a map of auditory space emerges (for review, see [10]). As mentioned earlier, NA and NL terminal fields in zebra finch MLd are not strictly segregated, but rather overlap substantially. These inter-species differences may reflect different evolutionary demands on the auditory system in these two lineages. Barn owls, as nocturnal hunters, excel at sound localization [74], [75], while zebra finches, as songbirds, perform rather poorly in sound localizing tasks [76] (but see also [77]). This difference in itself could suggest that the functional organization of MLd in songbirds is different from that in barn owls. In any case, exhaustive neurophysiological studies in the zebra finch midbrain are required to establish proper functional subdivisions. The few studies that have investigated electrophysiological properties of neurons in the finch MLd have reported a dorsoventral tonotopic organization of the nucleus, with low frequencies being represented dorsally and high frequencies ventrally [59], as seen in other avian species and in the mammalian central nucleus of the inferior colliculus [78]. By comparing the recording loci of Woolley and Casseday (Figure 3, p. 139, [59]) with the staining patterns of the present study, it seems that these recording loci fall outside the external edges of both our inner CR-positive region and our PV-positive region with less puncta, suggesting that they were located in what we call the outer region of MLd. This conclusion must remain hypothetical without a double labeling of recording loci and CR-IR. Thus, whether MLd.I and MLd.O exhibit different physiological properties remains to be established. Unlike the case in barn owls, these regions in finches are probably unrelated to the encoding of ITDs and ILDs, but rather to the processing of biologically relevant vocalization signals, given the differences in the organization of ascending inputs [19], [20]. Woolley and Casseday [59], [60] and Logerot et al. [79], described neurons in zebra finch MLd with tuning properties indicative of the processing of complex acoustic signals, especially their temporal aspects. Woolley et al. [61] reported four functional groups in the midbrain of the zebra finch, each of them being involved in the extraction of different features of vocal sounds. They identified broadband neurons, narrow-band temporal neurons, wideband neurons and two-band excitatory based on the spectrotemporal receptive fields of these neurons. Thus, in songbirds, feature extraction from songs might actually be a more important function than sound localization, and could be necessary for accurate song recognition [77], although the likelihood of single units coding for songs seems remote. Rather, synchronized responses of populations of neurons are proposed to “create a neural representation of the temporal patterns of the song” ([62] p. 2510). Precise recording localizations of Woolley and Casseday [59], [60] and Logerot et al. [79] units in respect to the boundaries we have defined in the present study would be a valuable addition in understanding the role of MLd subdivisions in the finch. In terms of population encoding, Poirier et al. [72] investigated MLd's responses to the Bird's Own Song (BOS), conspecific (CON) and heterospecific vocalizations. They found lateralized processing to BOS and CON, with BOS selectivity in the right MLd and CON selectivity in the left MLd. No differences were found in the present study between the staining patterns of left and right MLd. Using IEG immunocytochemistry, Woolley and Doupe [80] found no differences in ZENK expression in response to familiar or unfamiliar songs in MLd. This further adds to the necessity of investigating neuronal properties in MLd within the context of the anatomical subdivisions and how these relate to ascending inputs.

### Comparison with other tetrapods

In the present study we have deliberately eschewed a core and shell divisional schema for MLd of the zebra finch, because of potential interpretive problems associated with comparisons across species and authors: what is shell for some workers is core for others, and vice versa. Never the less, a core/shell (or belt) organization of the auditory midbrain is frequently assumed in the literature and is thought to be a conserved feature of tetrapods. The core is exclusively auditory by way of the topographic projections from lower brainstem auditory nuclei which result in a characteristic tonotopic organization of the nucleus. Moreover, the core conveys auditory information to the thalamus over lemniscal pathways. The belt is the site of ascending multimodal sensory information, including auditory, and receives descending projections from higher auditory centers.

In mammals, the core or central nucleus of the inferior colliculus (ICC) can be differentiated from the belt area (composed of dorsal (DC) and lateral (LC) cortices) on the basis of cytoarchitecture, auditory and somatosensory projections (for review see [81], [82]) or neurophysiological properties (for review see [83]). Numerous studies have shown the complementarity of parvalbumin and calbindin as well as calretinin staining, with PV-LI structures predominantly in the ICC and CR-LI and CB-LI structures predominant in the shell (bats: [54], [56]; chinchillas: [46]; gerbils: [51]; humans: [52], [53]; mice: [45], [55], [57]; rat: [41]–[43], [47]–[50]).

Similar observations have been reported in reptiles and amphibians, although the number of studies and species investigated are fewer. The torus semicircularis (auditory midbrain) of reptiles is composed of a central nucleus (Ce) –surrounded by laminar and superficial (L) nuclei. The cytoarchitecture, hodology and electrophysiological properties of these regions have been characterized [84]–[96]. The laminar nucleus has been proposed to correspond to part of the intercollicular nucleus of birds [97]. Belekhova et al., [37], [38] reported calcium-binding protein staining in the turtle midbrain. As is seen in mammals and birds, Ce was highly PV immunostained and CB-LI and CR-LI structures were mainly seen in the laminar nucleus. Interestingly, the staining patterns they observed in Ce were not homogeneous: the core region of the central nucleus (Cec) presented PV-LI staining while PV-, CB- and CR-LI structures were seen in the peripheral area of the central nucleus (Cep). Yan [39] and Yan et al. [40] investigated the pattern of staining in the midbrain torus of the gecko that revealed the presence of PV-, CB- and CR-LI structures in both the central and laminar nuclei. The staining patterns of these three CaBPs within the central nucleus showed distinct distributions, indicating subdivisions that could delineate some segregation of the ascending auditory projections. Whether a tonotopic organization of the gecko's torus exists is unknown, which makes it difficult to relate to the present findings in zebra finch. Clearly, detailed electrophysiological studies are needed before any clear conclusion can be drawn as to a similar organization of the reptilian torus and the mammalian and avian auditory midbrains.

In amphibians, three subdivisions of the torus semicircularis are recognized: principal nucleus (Tp), laminar nucleus (Tl) and magnocellular nucleus (Tmc). The morphology, ascending projection patterns and neurophysiological properties of these three areas are well documented [98]–[111]. As seen in sauropsids and mammals, there is an exclusively auditory area, Tp, and multisensory areas Tl and Tmc. Very few studies have reported patterns of calcium-binding protein immunoreactivity in the midbrain of amphibians and only the results for parvalbumin staining are available [35], [36]. In their study, Endepols et al. observed a clear cut boundary between Tp, which was strongly PV-positive and Tl, which was completely devoid of PV-LI. In Tmc, however, some PV-positive cell bodies were visible. Zeng et al. [36] reported that Tp neuropil showed a stronger parvalbumin staining than did Tl or Tmc.

These studies in various tetrapods show that the tonotopically organized, central nucleus is characteristically strongly immunostained and delineated with CaBPs. However, as we pointed out for birds, the existence of similar “anatomical” subdivisions cannot be taken to imply functional parallels between different species. First, as mentioned earlier, the pattern of auditory ascending projections in the zebra finch [19], [20] appears to be closer to that seen in mammals, in which there are topographically organized but overlapping terminal fields of brainstem projections (for review see [112]). But the regions defined by different CaBPs seen in the present study are somewhat closer to those described in the barn owl and chicken [3], [11], [12], [14], or in reptiles [37], where there are two distinct subdivisions within the central nucleus. These apparent mismatches between MLd subdivisions and the pattern of ascending auditory inputs raise the question of the functional significance of these differences in the zebra finch MLd. As stated above, NA and NL show overlapping terminal fields in both the inner and outer regions of MLd [19]. Contralateral and ipsilateral ascending projections of the LLV may, however, differentiate between these two regions (Figure S1). Ipsilaterally, LLV projections can be seen in both the inner and outer regions of MLd, while contralaterally, they appear to be restricted to the outer MLd (Materials and Methods S1; Figure S1). Biotinylated dextran amine injections into nucleus ovoidalis (Ov) also delineate both the inner and outer regions of MLd: retrogradely labeled somata are mainly present within MLd.I (Materials and Methods S1; Figure S2) but are also seen in MLd.O. Moreover, CB-LI and CR-LI structures defined a region just outside MLd that may correspond to the belt of other tetrapod ICs. However, our previous studies [19], [20] clearly showed that this region is not an apparent recipient of any ascending auditory brainstem projections and we therefore chose to classify this region as part of the ICo. Whether the outer region of MLd could be homologous to the belt region of other vertebrates cannot be ascertained because of the lack of studies focusing on somatosensory projections to the songbird auditory torus which could reveal an area in which multisensory inputs take place.

## Supporting Information

Figure S1 **A–D:** caudal (top) to rostral (bottom) transverse sections showing projections to the contralateral MLd after BDA injection in right LLV. Note the presence of ascending auditory fibers and terminal fields mainly in the outer region of MLd. **E–H:** caudal (top) to rostral (bottom) transverse sections showing projections to the ipsilateral MLd after BDA injection in right LLV. Note that the ascending auditory fibers and terminal fields can be observed in both the inner and outer region of MLd. Scale bars = 100 µm.
(TIF)

Figure S2 **A–D:** caudal (top) to rostral (bottom) transverse sections showing projections from the ipsilateral MLd after BDA injection in Ov. Note that retrogradely labeled somata are mainly visible and located within the inner MLd. Also note the absence of projections from the ICo. Scale bar = 100 µm. **E–H:** Schematic drawings of the different regions based on the PV-, CR- and CB-LI stainings from the present study.
(TIF)

Materials and Methods S1 Materials and Methods to accompany Figures S1 and S2.
(DOC)
