# Supplementary figures and images for: Subdivisions of the Auditory Midbrain (N. Mesencephalicus Lateralis, pars dorsalis) in Zebra Finches Using Calcium-Binding Protein Immunocytochemistry
Source: PLoS One. 2011 Jun 20;6(6):e20686. doi: 10.1371/journal.pone.0020686 (PMC3119058; doi:10.1371/journal.pone.0020686)

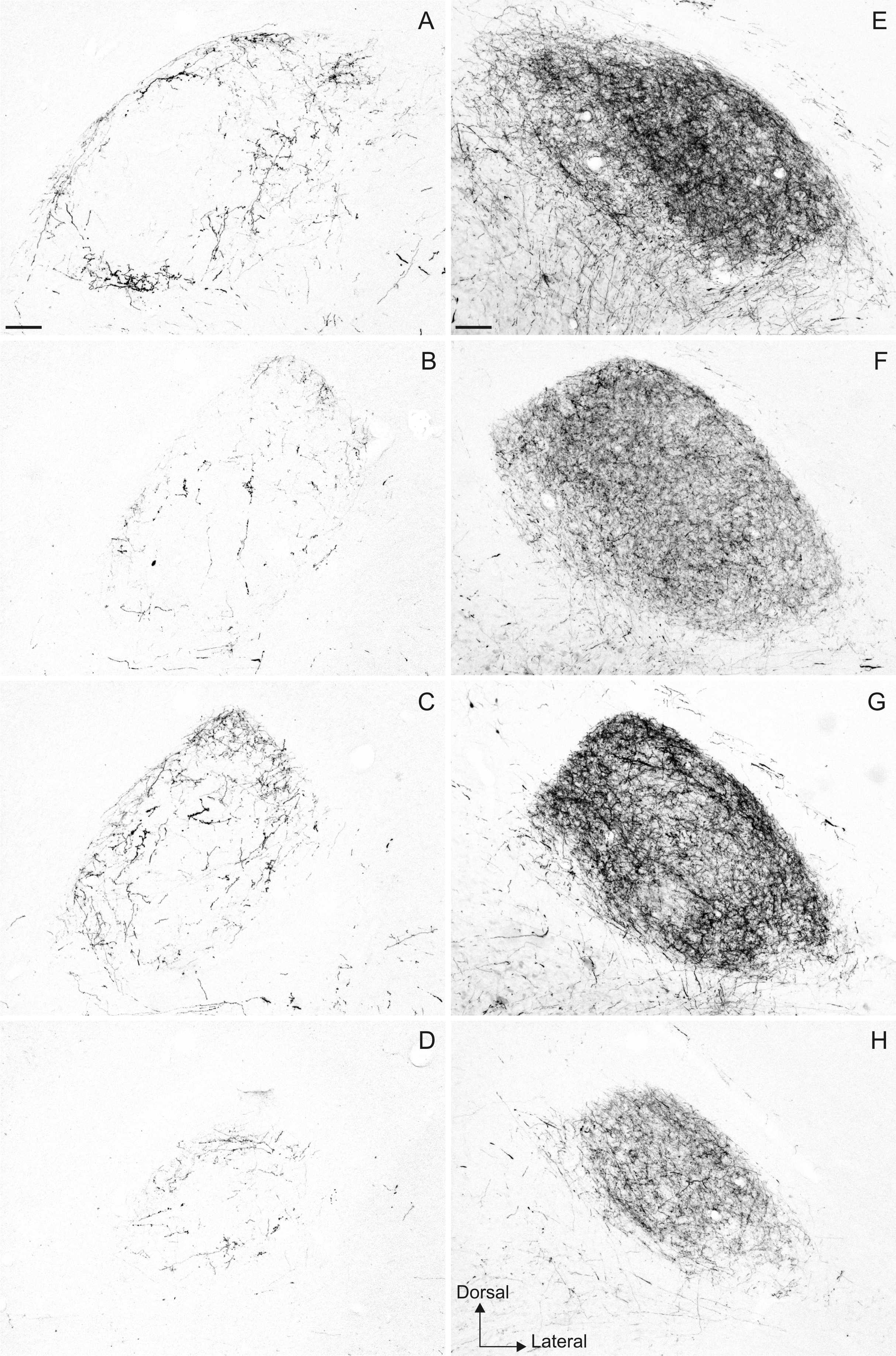

Supplement: Figure S1 — A–D: caudal (top) to rostral (bottom) transverse sections showing projections to the contralateral MLd after BDA injection in right LLV. Note the presence of ascending auditory fibers and terminal fields mainly in the outer region of MLd. E–H: caudal (top) to rostral (bottom) transverse sections showing projections to the ipsilateral MLd after BDA injection in right LLV. Note that the ascending auditory fibers and terminal fields can be observed in both the inner and outer region of MLd. Scale bars = 100 µm. (TIF) [file pone.0020686.s001.tif]

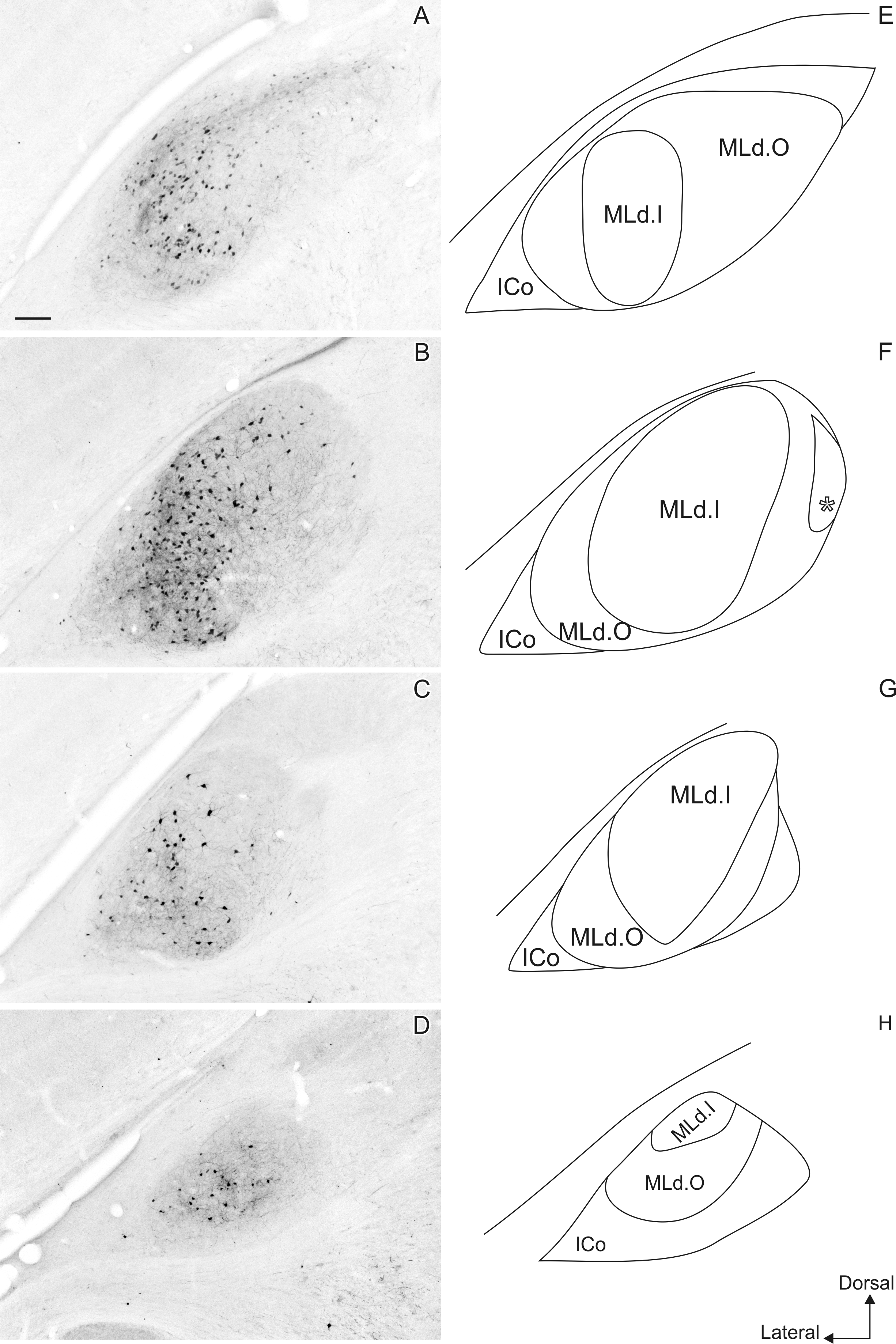

Supplement: Figure S2 — A–D: caudal (top) to rostral (bottom) transverse sections showing projections from the ipsilateral MLd after BDA injection in Ov. Note that retrogradely labeled somata are mainly visible and located within the inner MLd. Also note the absence of projections from the ICo. Scale bar = 100 µm. E–H: Schematic drawings of the different regions based on the PV-, CR- and CB-LI stainings from the present study. (TIF) [file pone.0020686.s002.tif]
