# Supplementary material for: Subdivisions of the Auditory Midbrain (N. Mesencephalicus Lateralis, pars dorsalis) in Zebra Finches Using Calcium-Binding Protein Immunocytochemistry
Source: PLoS One. 2011 Jun 20;6(6):e20686. doi: 10.1371/journal.pone.0020686 (PMC3119058; doi:10.1371/journal.pone.0020686)
Supplement: Materials and Methods S1 — Materials and Methods to accompany Figures S1 and S2. (DOC) [file pone.0020686.s003.doc]

**Materials and Methods S1**

**Biotinylated dextran amine injection into LLV and Ov**

Injection and immunocytochemistry protocols have been described in a previous publication [1]. In short, birds were anesthetized with an intramuscular injection of ketamine (100 mg/kg) and xylazine (20 mg/kg). They were placed in a stereotaxic apparatus (Kopf, Tujunga, CA), head tilted down 45º to the horizontal plane [2]. The injection site was determined by the use of electrophysiological identification of the auditory-responsive nuclei. Once the nucleus was identified, injection of biotinylated dextran amine (BDA; 10,000 MW, Invitrogen, Eugene, OR; 10% in 1 M NaCl in LLV; Molecular Probes, Eugene, OR in Ov) was made through glass micropipettes (outer diameter, 12-20 μm) by iontophoresis (2-4 μA positive current for 10-20 minutes). After a survival time of 3-4 days, birds were deeply anaesthetized and transcardially perfused with 0.9% saline followed by 4% paraformaldehyde in phosphate buffer (PB; pH 7.4). The brains were post-fixed in 4% paraformaldehyde before being cryoprotected in a 30% sucrose in phosphate buffered saline (PBS; pH 7.4). They were then cut coronally on a freezing microtome and serial, free-floating 35μm thick sections were collected in PBS and immediately processed for immunocytochemistry. After three 10 minutes rinses (all rinses in this protocol lasted 10 min) in PBS, endogenous peroxidase activity in the sections was blocked using 50% methanol and 1% hydrogen peroxide (H_2_O_2_) in distilled water for 10 min at room temperature. Sections were then rinses 3 times in PBS before being incubated in Neutravidin for LLV (Pierce Biotechnology, Rockford, IL) or Streptavidin for Ov (Molecular Probes, Eugene, OR) at 1:1,000 in PBS with 0.4% Triton-X for 1h at room temperature. Following 3 rinses in PBS, sections were finally incubated in a chromogen-solution (0.025% 3,3’-diamino-benzidine [DAB], 0.005 % H_2_O_2_ and 0.015% CoCl2). The reaction was stopped by several washes in PBS. Sections were subsequently mounted on subbed slides, dehydrated in successive ethanol baths, cleared in xylene, and coverslipped using DePeX.

**References**

1. Krützfeldt NOE, Logerot P, Kubke MF, Wild JM (2010) Connections of the auditory brainstem in a songbird, Taeniopygia guttata. I. Projections of nucleus angularis and nucleus laminaris to the auditory torus. The Journal of Comparative Neurology 518: 2109-2134.

2. Stokes TM, Leonard CM, Nottebohm F (1974) The telencephalon, diencephalon, and mesencephalon of the canary, Serinus canaria, in stereotaxic coordinates. J Comp Neurol 156: 337-374.
